# COVID-19: The Great Reset – A Review: Review of Klaus Schwab and Thierry Malleret, *Covid-19: The Great Reset* (Forum Publishing, 2020), 280 pages, $10.22

**DOI:** 10.1007/s42413-021-00117-7

**Published:** 2021-05-10

**Authors:** Peter B. Meyer, Reimund Schwarze

**Affiliations:** 1 University of Louisville, Louisville, KY USA; 2 The E.P. Systems Group, New Hope, PA 18938 USA; 3 Helmholtz Centre for Environmental Research — UFZ, Leipzig, Germany

The COVID-19 pandemic has challenged the socioeconomic system of modern capitalism at a level comparable to World War II and can be expected to reshape the system as extensively. There is no going back to the way we were; we need a “great reset,” but the question is of what type. Klaus Schwab, founder of the World Economic Forum and senior author of this volume, advocates a stakeholder capitalism to replace today’s financial capitalism. Despite its title, this book does not offer a guide to attaining the reset he proposes but rather lays out alternative forms the reset may take.

While it challenges traditional economic principles, it follows the discipline in its argument, beginning with a long discussion of the macro reset – the global choices – moving from economic to social, geopolitical, environmental and technological resets, followed by the micro reset, choices firms must take and then individual reset, basically discussion of consumer behavior. The community as a unit of socioeconomic activity is ignored although some sense of community and its well-being shape all responses to the pandemic.

Schwab and Malleret argue that the macro reset will be shaped by three intersecting facets of the current system: Interdependence or systemic connectivity, velocity and complexity, all of which have grown rapidly. The first involves supply chains and information linkages and affects the spread of infection and information. The second has made time a “scarcity element” and generated volatility in financial markets and voting behavior as all actors become more impatient. The third is a characteristic of any complex adaptive system, including both the pandemic and the socioeconomic system it is impacting. The three combine to generate “quantum politics” with the unpredictability and uncertainty that makes the pandemic itself create a “window of opportunity” for constructive system change.

The economic reset will have to start from the deepest crisis in industrial history. What began as a local epidemic in Wuhan in December 2019 has developed into a global pandemic along deeply interconnected trade networks. The necessary closures have led to GDP slumps of between 20–30% in in the industrialized countries. For comparison consider that the global economic crisis of the 1920s and the 2008/2009 financial crisis each led to a decline in economic output of under 10%. The International Labour Organization estimates the worldwide loss of full-time jobs at between 140 and 340 million. Most affected are informal workers and people in low-paid jobs who were living with greater risk of illness and lower job security before the pandemic hit. If GDP continues to fall at the current rates, up to 580 million people worldwide would be pushed into extreme poverty. This results not only in hardship and existential fears for those affected, but also makes it difficult—if not impossible—for them to follow lockdown orders and comply with other pandemic prevention measures. Wealth and health during pandemics must be considered as complementary goods. Far from the perception of the pandemic as the "great leveller", the COVID 19 crisis has clearly reinforced the segregation of labor markets, poverty and disease both within and between countries.

These threatening inequalities are truly shocking. That is why Schwab and Malleret promote a strategy of inclusive recovery. The central elements of this program include: Cost-effective health care systems around the world, social safety nets, extended unemployment benefits, communities with high levels of trust, a real sense of solidarity and overcoming today’s incredible divide between the market value and the societial value of work. They argue that society in the future “will hardly accept that a star hedge fund manager who specializes in short-selling … can receive an income in the millions per year while a nurse … earns infinitesimal fraction of that amount" (p. 83). The alternative to this program would be social unrest.

COVID19 and the climate crisis have five things in common: Both are systemic risks; they are non-linear; the distributional impacts are harsh; they are global in nature and they affect countries and people disproportionately. But important differences remain: the time horizon and the causality problem. The Corona crisis itself cannot contribute to leading the world into a low-carbon, green and resilient future. Even though the crisis in March and April 2020 resulted in global carbon dioxide emissions reductions of up to 30% and some heavily polluted cities such as New Delhi finally reported "blue skies" again, this cannot hide the fact that pandemic and the measures taken to combat it will not reverse the climate change trend.

Schwab and Malleret therefore advocate a bold reorientation after the shutdown. The situation is critical in their view: the global stimulus packages could help drive economic development towards low-carbon and climate-resilient development or lock us into long-term development paths that increase climate risks and the vulnerability of the world (p. 143). Chances for a green recovery are good: The departure from the previously dominant demand stimulation to investment stimulation in most national economic stimulus packages can already be seen as a success. Moreover, it has now become undisputed among economists that "green" research and infrastructure spending is significantly more employment-promoting in the long term than general ("grey") research and infrastructure spending. But a green recovery needs more political than good economic arguments. Schwab and Malleret focus on four trends they expect to drive the world to a greener future:: enlightened leadership; growing risk awareness; pandemic-driven behavior change, and growing local and national activism.

Reflecting on these trends, we can note that the US has not enjoyed enlightened leadership but this may change in the coming year while Europe and the much of rest of the world appear to tecognize that a green recovery will best serve their economic as well as political needs. This trend is in part due to the increased risk awareness exemplified by the increased investments by major fossil fuel companies and financial institutions in renewable energy projects Demand for fossil fuels for travel has fallen precipitously during the pandemic as has demand for material goods relative to services. Both declines may reflect behavior changes that presage increased future reliance on community networks to serve needs and lower greenhouse gas emissions. These pandemic-driven changes are strengthened and reinforced by local efforts to protect communities from economic devastation and concern over environmental pollution.

Schwab and Malleret thus consider the pandemic to offer a “window of opportunities” for a new and better future. While they acknowledge that the pandemic could depress financial returns for up to 40 years, it also has demonstrated that the putative tradeoff of health or environment protection versus economic activity stimulation is false. Increased reliance on labor substitution as a tool for health protection threatens jobs, but may also force a reconsideration of the how the gains from economic activity are distributed. The high unemployment and decline in small business activity have already forced countries to provide financial support unrelated to work to their citizens. This, they believe, leads to new missions for fiscal and monetary policy in providing economic and social security to citizens.

At the level of the firm, which is where most economic activity occurs, Covid-19 has driven a change from a “just-in-time” production logic to one of developing “just-in-case” resilience capacity. This is the window through which what they call stakeholder capitalism will emerge as the guiding business principle. Evidence suggests this may already be occurring as companies guided by environmental, social and governmental (ESG) principles have outperformed their rivals during the pandemic and funds managed on the principles are attracting more money from investors.

The hope for a constructive reset thus emerges from the expectation that “the pandemic has acted as a dramatic eye-opener to the public at large on the severity of the risks related to environmental degradation and climate change.” (p. 238) They note, however, that the hope must be tempered by the reality that the “absolute prerequisite for a proper reset is greater collaboration and cooperation within and between countries.” (p. 248) The reset, then relies on changes in individual perceptions and priorities, but those individual objectives cannot be attained without new and better trans-national relations. How those relations can be transformed remains uncertain and the authors offer no more than hope.

Hope may, however, come from another source. The needed leadership transcending national interests appears to be emerging from the institutional investment sector. Preservation and growth of asset values are important to investors and they are beginning to recognize the risks that climate change and other environmental challenges may pose. In September, 2020, Climate Action 100 + , a cooperative initiative of over 500 institutional investors controlling over $47 Trillion, issued a warning to 161 fossil fuel, mining, transport and other high greenhouse gas emitting companies that investments in them will be assessed relative to 30 climate measures and targets beginning in early 2021. If those investors shift their assets toward greening the planet, local communities may get improved access to the funds they need to act. Schwab and Malleret may be proven correct in their predictions, but for the wrong reasons.

“*Covid-19: The Great Reset* is a book of big words, of sharp social accusations and of ambivalent hope; a book that appeals to political morality rather than really tracing the “mechanics of the pandemic”; and a projection taking great hope from the social activism of oppressed minorities and youth. It is a book that will find a broad readership—despite its weaknesses—because it offers hope for the system of modern capitalism by transforming its logic to build back better. 

